# A Positioning Error Compensation Method for a Mobile Measurement System Based on Plane Control

**DOI:** 10.3390/s20010294

**Published:** 2020-01-04

**Authors:** Bo Shi, Fan Zhang, Fanlin Yang, Yanquan Lyu, Shun Zhang, Guoyu Li

**Affiliations:** 1 College of Geomatics, Shandong University of Science and Technology, Qingdao 266000, China; shibo@sdust.edu.cn (B.S.); fanzhang@sdust.edu.cn (F.Z.); yanquanlv@sdust.edu.cn (Y.L.); shunzhang@sdust.edu.cn (S.Z.); 2 Qingdao Xiushan Mobile Surveying Co., Ltd., Qingdao 266000, China; liguoyu@supersurs.com

**Keywords:** position and orientation, plane feature, Gaussian model, laser point cloud

## Abstract

Global navigation satellite system (GNSS)/inertial navigation system (INS) navigation technology is one of the core technologies in a mobile measurement system and can provide real-time geo-referenced information. However, in the environment measurements, buildings cover up the GNSS signal, causing satellite signals to experience loss-of-lock. At this time errors of INS independent navigation accumulate rapidly, so it cannot meet the needs of the mobile measurement system. In this paper, a positioning error compensation method based on plane control is proposed by analyzing the error characteristics of position and orientation caused by satellite signal loss-of-lock in the urban environment. This method control uses planar features and the laser point cloud positioning equation to establish an adjustment model that ignores the influence of the attitude error and finds the positioning error at the middle point of the GNSS signal loss-of-lock time period, and then compensates for the error at other points by using the characteristics of the positioning error. The experimental results show that the accuracy of the compensated laser point cloud has been significantly improved, and the feasibility of the method is verified. Meanwhile, the method can rely on the existing building plane so the method is adaptable and easy to implement.

## 1. Introduction

A mobile mapping system (MMS) integrates measurement sensors such as a position and orientation system (POS), a laser scanning system and a charge-coupled device (CCD) camera on a mobile carrier [1,2], enabling the fast and efficient acquisition of spatial data on surrounding targets, which is widely used in three-dimensional modeling, digital city, street view map, digital mine and other fields [3,4,5,6]. In the measurement process, MMS needs the high-precision position and orientation information obtained by POS to realize the conversion from a laser scanner coordinates system to the specified mapping coordinate system. At present, the POS of the mobile measurement system mainly adopts the Global Navigation Satellite System (GNSS)/inertial navigation system (INS) combined navigation [7]. When MMS is measured in an urban environment, GNSS signals can easily be blocked by the tall buildings in the city which cause the satellite signals to experience loss of lock. An integrated navigation system can only rely on INS independent navigation. Since INS is a continuous integration system, if navigation is performed for a long time, the inertial device errors will continue to accumulate. In this case, the POS system cannot provide accurate position and orientation information, and the accuracy of the laser scanning system will be reduced from the sub-meter to the meter level [8,9].

Domestic and foreign researchers have conducted a great deal of research on how to improve the accuracy of MMS during satellite signal loss-of-lock. The research results can be summarized into two strategies: correcting POS information by means of constraints and smoothing, or compensating for laser point cloud accuracy with feature points. The former fixes the navigation data error by adding constraints and constructing a filter equation of additional motion constraints by using the motion law of the carrier [10,11,12]. However, this method usually constructs constrained observation vectors with fixed observations. The actual observation value is not a constant, and is greatly affected by topographical and carrier motion changes, so the navigation accuracy after improvement cannot be guaranteed. The performance of the GNSS/INS integrated navigation system can also be improved by adding auxiliary sensors such as odometers, visual sensors, and LiDAR (light detection and ranging) to compensate for the INS errors [13,14]. However, the initial value of the odometer scale might be affected by temperature and tire pressure in this method is not clear, the error of the odometer scale accumulates linearly with driving distance and is greatly affected by the speed of the vehicle. The visual sensor is susceptible to light and so we cannot guarantee POS accuracy. LiDAR is less affected by the environment and is inexpensive, so it is a sensor suitable for positioning assistance. However, this method mostly uses a Kalman filter (KF) or other filter models to fuse the navigation information of LiDAR and INS to achieve positioning and orientation. Among them, for LiDAR, the estimation of carrier position and attitude is mainly obtained by scanning matching on continuously-scanned images. When there is no overlapping area between consecutively-scanned images or the image surface geometry is not good, it will seriously affect the accuracy of scanning matching and lead to a significant reduction in POS accuracy. In addition, in the post-processing of GNSS/INS combined navigation data, smoothing is used to improve the accuracy of combined navigation [15,16,17,18,19,20]. The smoothing process can directly correct the POS error in the loss-of-lock period by directly using the GNSS data from before and after without adding other observations. The effect of improving the attitude error after smoothing is significant, but positioning error increases with satellite signal loss-of-lock time, so it is difficult to ensure the accuracy of the MMS measurement. It is possible to obtain an external positioning update at a given time by calculating the coordinate difference between the pre-deployed feature points with precise coordinates and the coordinate points of the same-named feature points in the point cloud [21,22]. Feature points can also be extracted within the trajectory, and the improved Iterative Closest Point (ICP) algorithm is used to analyze the incorrect correspondence of points. Then the least squares method is used to calculate the corrected position and attitude of the trajectory feature points. Finally, the positional rotation parameters of the corresponding trajectory points are calculated by linear interpolation [23]. However, this method requires a large number of feature points to be set and requires precise feature points calculated by the traditional method, which has a significant workload and low efficiency. Moreover, extracting feature points of the same name mainly relies on manual operation. The accuracy of the extraction is highly correlated with the point cloud density. In addition, this method can only correct the error of the laser point cloud; it does not improve the accuracy of the POS, and it is difficult to obtain accurate trajectory information, so the method has certain limitations in terms of its practical application.

In this paper, a positioning error compensation method based on plane control is proposed. The method makes full use of the geographical environment in the satellite loss-of-lock area and uses the existing building plane as the control plane to calculate the positioning error at an intermediate time. Based on the analysis of characteristics for the positioning error after smoothing, the positioning error at other times can be compensated for. This not only avoids the difficulty of feature point extraction and the inaccuracy of extraction, but also obtains accurate track information. In addition, the method overcomes the limitations of the hardware and environment brought about by constraint conditions and auxiliary sensors. At the same time, it improves the work efficiency in practical applications.

## 2. Analysis of Position and Orientation Error Characteristics

**Figure 1:**
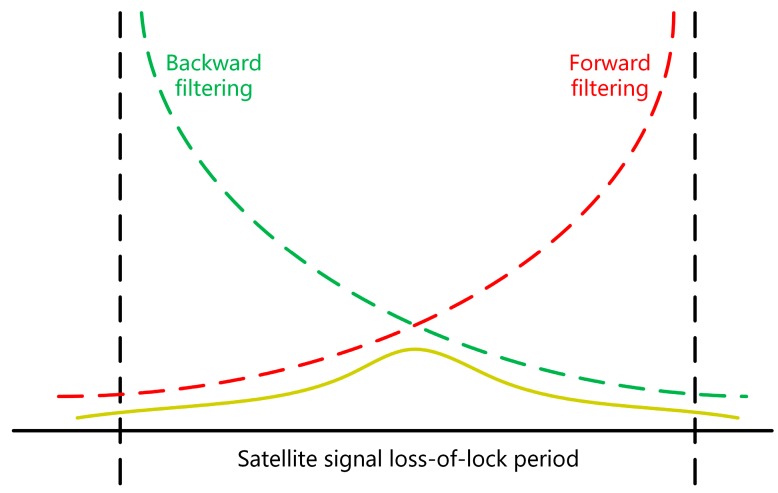
Smooth schematic diagram during the satellite signal loss-of-lock.

In this paper, the proposed method compensates for the POS error by using the relationship between the smoothed POS error and the GNSS loss-of-lock time. Therefore, the characteristics of the smoothed POS error need to be analyzed. Taking a traditional forward-backward smoothing filtering algorithm as an example, the forward and backward filtering achieve smooth estimation of the period loss-of-lock of satellite signals by using the data before and after the loss of the GNSS signal [24], as shown in Figure 1.

**Figure 2:**
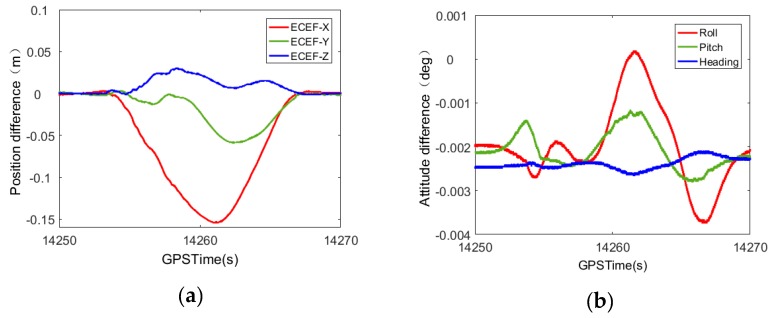
Comparison of POS error after 1-min smooth solution of satellite signal loss of lock. (**a**) Position error comparison; (**b**) attitude error comparison.

**Figure 3:**
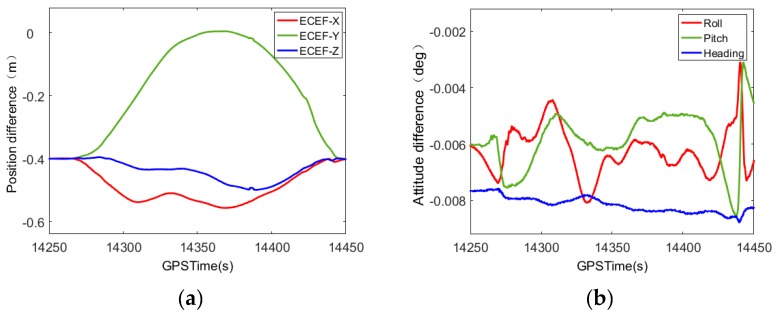
Comparison of POS errors after 3-min smooth solution of satellite signal loss-of-lock. (**a**) Position error comparison; (**b**) attitude error comparison.

Figure 1 shows that the smoothed results present a trend of first rising and then falling, similar to the shape of the Gaussian function distribution. The related literature research has yielded similar results [15,16,17,18,19]. This article uses a set of measured data from a mobile measurement system as an example. Navigation data were calculated using IE8.6, Novatel’s commercial software (The software is manufactured by Novatel Corporation of Alberta, Canada.), and GNSS satellite signals were set to cover a loss-of-lock of 1 min or 3 min. The results were different from those obtained without the loss-of-lock. The results are shown in Figure 2 and Figure 3, and the position and attitude error data are shown in Table 1.

The satellite loss of lock lasts 1 min, as shown in Figure 2.

The satellite loss of lock lasts for 3 min, as shown in Figure 3.

From the trend of the curve in Figure 2 and Figure 3, it can be seen that, after the smoothing process, the attitude error has a certain fluctuation. The error value is always within a thousandth of a degree, which meets the requirements of the mobile measurement accuracy. The positioning error shows a trend of increasing from zero to the maximum error and then decreasing from the maximum error to zero in the satellite loss-of-lock period. This trend is consistent with the shape proposed in this paper, which is similar to the Gaussian function distribution. When the GNSS signal experiences loss-of-lock for 1 min, the positioning error is still on the decimeter level. Although it definitely experiences an improvement, it still cannot meet the needs of mobile mapping.

## 3. Positioning Error Compensation Based on Plane Control

**Figure 4:**
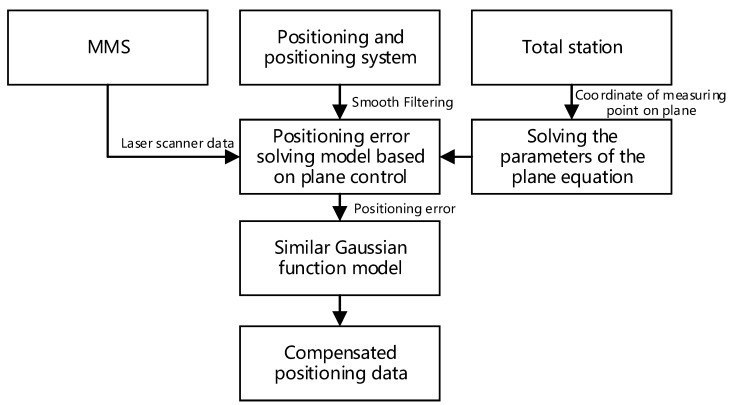
Positioning error compensation based on plane control.

The positioning accuracy cannot meet the needs of mobile mapping after the smoothing solution. In order to improve the positioning accuracy, this paper proposes a compensation method based on plane control. In this paper, the positioning error at the intermediate moment of GNSS loss-of-lock was first calculated in the area where the GNSS signal loss-of-lock using the control plane. Then, according to a relationship similar to a Gaussian function between the smoothed positioning error and the GNSS loss-of-lock time, the positioning error at other times is compensated for. As shown in Figure 4, first of all, a total station is used to measure the coordinates of 15–30 measuring points which are evenly distributed on the space plane, and then the space plane equation of the plane is calculated. At the same time, the laser point cloud data of the same plane measured by the mobile measurement system and the smoothed position and attitude information are used to construct the laser point cloud positioning equation. Then, Based on the spatial plane equation and the laser point cloud positioning equation, a position error solving model based on plane control is established. Finally, by analyzing the characteristics of the smoothed position error and using the calculated position error to construct a Gaussian function error compensation model, the position error is compensated.

### 3.1. Laser Point Cloud Localization Equation

The basic steps of a mobile measurement system positioning are based on the polar diameter and angle information of the target point measured by the laser scanner. It is the first to calculate the coordinates of the target point in the laser scanner coordinate system, and then goes through a series of coordinate transformations to achieve the conversion of the target point from a laser scanner coordinate system to the WGS-84 coordinate system. The positioning equation of the laser point cloud in the WGS-84 coordinate system is:

(1) $${\left[\begin{array}{c} X\\ Y\\ Z \end{array}\right]}_{WGS84}={\left[\begin{array}{c} X\\ Y\\ Z \end{array}\right]}_{oe}+R_{l}^{e}R_{b}^{l}\left[R_{s}^{b}{\left[\begin{array}{c} X\\ Y\\ Z \end{array}\right]}_{s}+\left[\begin{array}{c} l_{x}\\ l_{y}\\ l_{z} \end{array}\right]\right],$$

where ${\left[X,Y,Z\right]}_{S}^{T}$ is the point cloud coordinate in the laser scanner frame; ${\left[l_{x},l_{y},l_{z}\right]}^{T}$ is the amount of translation of the laser scanner frame to the body frame; $R_{s}^{b}$ is the rotation matrix of the laser scanner frame to the body frame; $R_{b}^{l}$ is the rotation matrix of the body frame to the local level frame; $R_{l}^{e}$ is the rotation matrix of the local level frame to the WGS-84 coordinate frame; ${\left[X,Y,Z\right]}_{\mathrm{oe}}^{T}$ are the coordinates of the local horizontal coordinate origin in the WGS-84 coordinate frame; and ${\left[X,Y,Z\right]}_{e}^{T}$ is the coordinates of the laser point cloud in the WGS-84 coordinate frame.

### 3.2. Positioning Error Solving Principle

The errors that affect the measurement accuracy of the mobile measurement system can be divided into three major components: POS error, laser scanner measurement error, and placement error after the integration of each sensor. With the development of laser scanner hardware, we must ensure that the measuring accuracy of the point position meets the specific measurement needs. The placement error between each sensor can reduce the measurement error by calibration. Therefore, the method in this paper ignores the influence of the laser scanner measurement error and the placement error after the integration of each sensor. Equation (1) can be expressed as follows:

(2) $${\left[\begin{array}{c} X\\ Y\\ Z \end{array}\right]}_{WGS84}={\left[\begin{array}{c} X\\ Y\\ Z \end{array}\right]}_{oe}+R_{l}^{e}R_{b}^{l}{\left[\begin{array}{c} X\\ Y\\ Z \end{array}\right]}_{b},$$

The POS error includes three positioning errors $\Delta X_{\mathrm{oe}}$, $\Delta Y_{\mathrm{oe}}$, $\Delta Y_{\mathrm{oe}}$ and three attitude errors Δr, Δp, Δy, which are added to Equation (2) to obtain the POS error:

(3) $${\left[\begin{array}{c} X\\ Y\\ Z \end{array}\right]}_{WGS84}={\left[\begin{array}{c} X\\ Y\\ Z \end{array}\right]}_{oe}+\Delta {\left[\begin{array}{c} X\\ Y\\ Z \end{array}\right]}_{oe}+R_{l}^{e*}\times \left(I+\Omega_{l*}^{l}\right)R_{b}^{l*}\times {\left[\begin{array}{c} X\\ Y\\ Z \end{array}\right]}_{b},\;\;\Omega_{l*}^{l}=\left[\begin{array}{ccc} 0 & \Delta y & \Delta r\\ -\Delta y & 0 & -\Delta p\\ -\Delta r & \Delta p & 0 \end{array}\right],$$

in which $R_{l}^{e*}$ is the rotation matrix of the local level frame to the WGS-84 coordinate frame when there is a positioning error; $\Omega_{l*}^{l}$ is the attitude error matrix; and $R_{b}^{l*}$ is the rotation matrix of the body frame to the local level frame when there is an attitude error.

Through the propagation of error, the error model of Equation (3) can be obtained:

(4) $$\left\{\begin{array}{l} \sigma_{x}=[{\left(\frac{\partial X}{\partial r}\right)}^{2}\sigma_{r}^{2}+{\left(\frac{\partial X}{\partial p}\right)}^{2}\sigma_{p}^{2}+{\left(\frac{\partial X}{\partial y}\right)}^{2}\sigma_{y}^{2}+{\left(\frac{\partial X}{\partial X_{oe}}\right)}^{2}\sigma_{X_{oe}}^{2}+{\left(\frac{\partial X}{\partial Y_{oe}}\right)}^{2}\sigma_{Y_{oe}}^{2}{+{\left(\frac{\partial X}{\partial Z_{oe}}\right)}^{2}\sigma_{Z_{oe}}^{2}]}^{\frac{1}{2}}\\ \sigma_{Y}=[{\left(\frac{\partial Y}{\partial r}\right)}^{2}\sigma_{r}^{2}+{\left(\frac{\partial Y}{\partial p}\right)}^{2}\sigma_{p}^{2}+{\left(\frac{\partial Y}{\partial y}\right)}^{2}\sigma_{y}^{2}+{{\left(\frac{\partial Y}{\partial X_{oe}}\right)}^{2}\sigma_{X_{oe}}^{2}+{\left(\frac{\partial Y}{\partial Y_{oe}}\right)}^{2}\sigma_{Y_{oe}}^{2}+{\left(\frac{\partial Y}{\partial Z_{oe}}\right)}^{2}\sigma_{Z_{oe}}^{2}]}^{\frac{1}{2}}\\ \sigma_{Z}=[{\left(\frac{\partial Z}{\partial r}\right)}^{2}\sigma_{r}^{2}+{\left(\frac{\partial Z}{\partial p}\right)}^{2}\sigma_{p}^{2}+{\left(\frac{\partial Z}{\partial y}\right)}^{2}\sigma_{y}^{2}+{{\left(\frac{\partial Z}{\partial X_{oe}}\right)}^{2}\sigma_{X_{oe}}^{2}+{\left(\frac{\partial Z}{\partial Y_{oe}}\right)}^{2}\sigma_{Y_{oe}}^{2}+{\left(\frac{\partial Z}{\partial Z_{oe}}\right)}^{2}\sigma_{Z_{oe}}^{2}]}^{\frac{1}{2}} \end{array}\right\}$$

In this equation, $\sigma_{X},\sigma_{Y},\sigma_{Z}$ are the mean square errors of the laser point cloud in the X, Y, and Z directions, respectively, in the WGS-84 coordinate frame; $\sigma_{r}^{2},\sigma_{p}^{2},\sigma_{y}^{2}$ are the mean square errors of the roll angle, pitch angle, and heading angle, respectively, and $\sigma_{X_{oe}}^{2},\sigma_{Y_{oe}}^{2},\sigma_{Z_{oe}}^{2}$ are the mean square errors of the positioning in the X, Y, and Z directions, respectively, in the WGS-84 coordinate frame.

According to the official data provided by Novatel, after IE8.6 software processing, the mean square error of the pitch angle and roll angle is 0.005°, the mean square error of heading angle is 0.008°, the mean square error of the horizontal direction is 0.01 m, and the mean square error of the vertical direction is 0.015 m when the satellite signal is not loss-of-lock. The mean square error of pitch angle and roll angle is 0.006°, the mean square error of the heading angle is 0.006°, the mean square error of the heading angle is 0.010°, the mean square error of the horizontal direction is 0.011 m, and the mean square error of the vertical direction is 0.03 m when the satellite signal loss-of-lock lasts for 60 s.

It can be seen that as the satellite signal loses lock time increases, the positioning error increases significantly, while the attitude error varies little. In addition, when the error model is used to calculate the accuracy of the laser point cloud, the effect of the positioning error on the accuracy of the laser point cloud is much greater than the attitude error. The attitude error can be smoothed to meet the needs of movement measurement. Therefore, the influence of attitude error is not considered in the calculation, so Equation (3) can be simplified as follows:

(5) $${\left[\begin{array}{c} X\\ Y\\ Z \end{array}\right]}_{WGS84}={\left[\begin{array}{c} X\\ Y\\ Z \end{array}\right]}_{oe}+\Delta {\left[\begin{array}{c} X\\ Y\\ Z \end{array}\right]}_{oe}+R_{l}^{e}\times R_{b}^{l*}\times {\left[\begin{array}{c} X\\ Y\\ Z \end{array}\right]}_{b},$$

The general form of the space plane is:

(6) ax+by+cz−d=0,

where a,b,c is the unit normal vector of the plane and d is the distance from the origin of the coordinate to the plane. Then the reference plane parameter a,b,c,d can be obtained using the eigenvalue method [25].

The laser point cloud data on the control surface satisfy the plane equation of the control surface, so:

(7) $$\left[\begin{array}{ccc} a_{p} & b_{p} & c_{p} \end{array}\right]{\left[\begin{array}{c} X\\ Y\\ Z \end{array}\right]}_{e}-d_{p}=0,\left[\begin{array}{ccc} a_{p} & b_{p} & c_{p} \end{array}\right]\left[{\left[\begin{array}{c} X\\ Y\\ Z \end{array}\right]}_{oe}+\Delta {\left[\begin{array}{c} X\\ Y\\ Z \end{array}\right]}_{oe}+R_{l}^{e}\times R_{b}^{l*}\times {\left[\begin{array}{c} X\\ Y\\ Z \end{array}\right]}_{b}\right]-d_{p}=0$$

In Equation (7), $a_{p}$, $b_{p}$, $c_{p}$, $d_{p}$ represent the plane parameter of the p-th control plane. ${\left[\begin{array}{ccc} X & Y & Z \end{array}\right]}_{b}^{T}$ is the point cloud data coordinates in the body frame. ${\Delta [\begin{array}{ccc} X & Y & Z \end{array}]}_{oe}$ is the obtained positioning error.

### 3.3. Similar to the Gaussian Distribution Function Error Compensation Model

**Figure 5:**
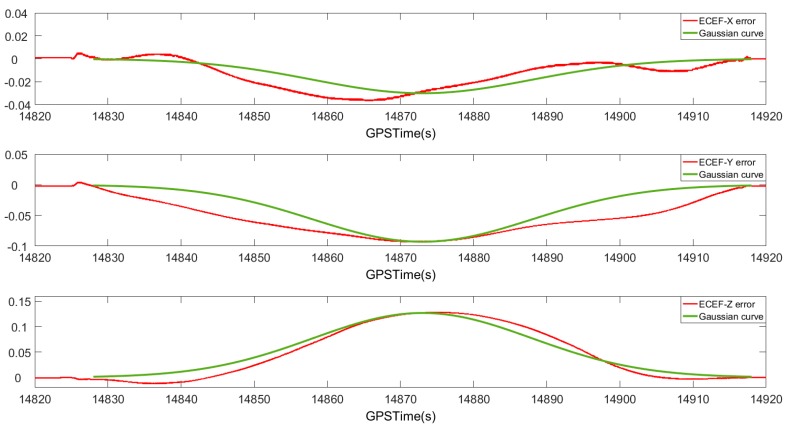
Comparison of position error curves and Gaussian curves after GNSS signal loss-of-lock.

By analyzing the characteristics of the positioning error after smoothing, it can be observed that the relationship between the positioning error after smoothing and time is similar to the Gaussian function feature. Taking the GNSS signal loss-of-lock for 90 s as an example, the relationship between the smoothed position error curve and the Gaussian function curve is shown in Figure 5. The red curve is the smoothed positioning error curve, and the green curve is the Gaussian function curve. It can be seen that the smoothed position error curve is similar to the Gaussian function curve.

It is assumed that the satellite signal is occluded at time $t_{0}\sim t_{n}$ and the navigation information calculated after smoothing reaches the maximum value at the intermediate time $t_{mid}$. The error and time in the satellite loss-of-lock period have the following Gaussian relationship:

(8) $$\begin{array}{l} y=b\times e^{\frac{-{\left(t-t_{mid}\right)}^{2}}{2\sigma^{2}}}\\ \sigma =\frac{\left(t_{n}-t_{0}\right)}{6} \end{array}$$

where b is the positioning error value at time $t_{mid}$, which can be obtained by the above method. The positioning error at other time points is calculated by a model similar to the Gaussian function, thereby compensating for the positioning error of the entire satellite signal during the loss-of-lock period.

## 4. Experimental Analysis

In order to verify the accuracy of the positioning error compensation method based on plane control, this paper chooses a VSurs-E mobile measurement system as the experimental platform. The feasibility of this method is demonstrated by comparing the positioning error before and after compensation and the accuracy of the laser point cloud.

### 4.1. Experimental Platform and Control Plane Selection

**Figure 6:**
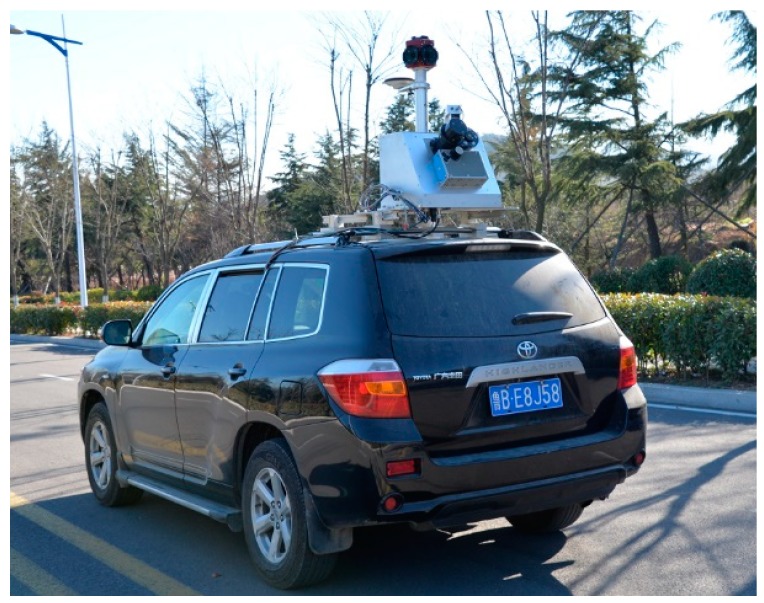
VSurs-E mobile measurement system.

The VSurs-E road information collection and inspection system was jointly developed by Shandong University of Science and Technology and Qingdao Xiushan Mobile Measurement Co., Ltd., as shown in Figure 6. The system is composed of Z+F PROFILER 9012 laser scanner (The Z + F Profiler 9012 manufactured by Zoller & Fröhlich GmbH Corporation of Wangen im Allgäu, Germany), a SPAN-LCI (The equipment is manufactured by Novatel Corporation of Alberta, Canada.) integrated navigation system, industrial cameras and other sensors. The system uses a car as a carrier to quickly collect three-dimensional spatial data such as road facilities and road topography. The parameters of VSurs-E’s laser scanner and integrated navigation system are given in Table 2.

**Figure 7:**
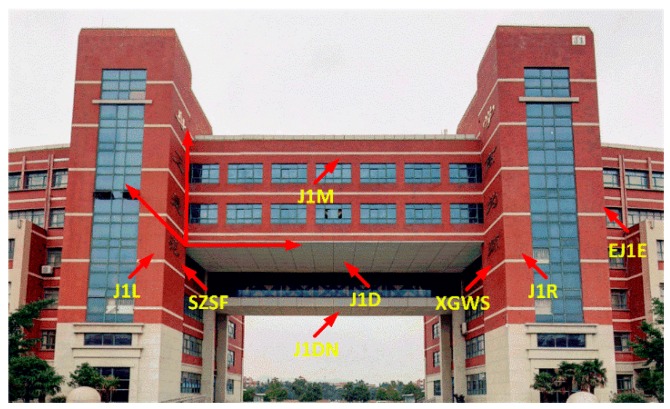
The J1 teaching building experimental area control plane.

**Figure 8:**
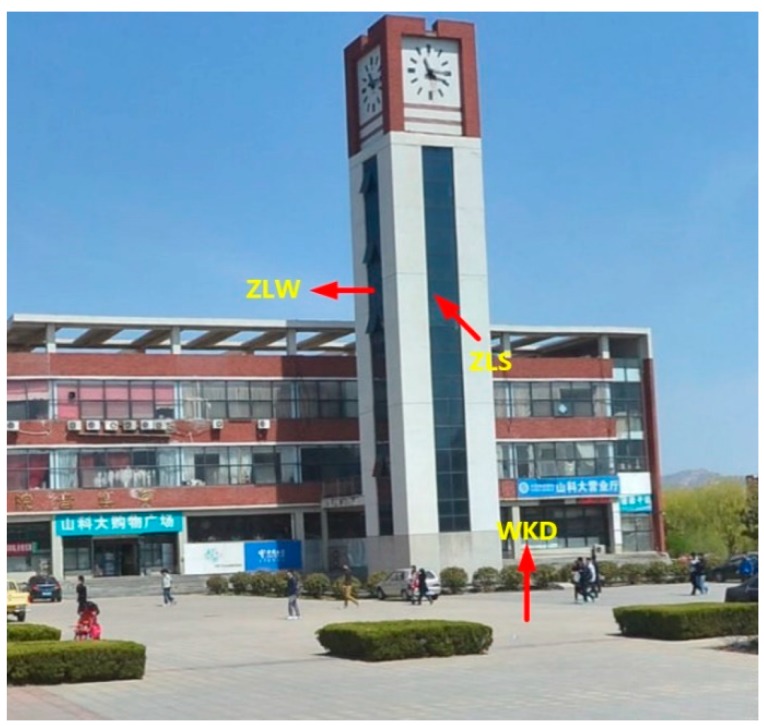
The restaurant experiment area control plane.

When the vehicle mobile measurement system is measured in an urban canyon environment, it can collect rich plane features, and there is a rich plane in the teaching building area. Therefore, the teaching building area is selected as the experimental site. In the J1 teaching building area, a total of seven groups of planes, which are the building’s smooth wall surfaces different orientations are selected as the control plane as shown in Figure 7. In addition, in the school restaurant, three planes with flat surfaces and different orientations are selected as the control plane as shown in Figure 8. The measurement of the experimental sites with the VSurs-E mobile measurement system and the reference planes were accurately extracted from the laser point cloud data. At the same time, based on accurate measurement of the control points, a high-precision total station is used to measure 15–30 points for each reference plane. These acquired data points provide data support for the parameters that compute the plane equations.

### 4.2. Positioning Error Simulation Analysis

In order to verify the accuracy of the method in this paper and analyze the influence of attitude error on positioning error, a simulation experiment was performed by using a set of navigation data that did not involve loss-of-lock. Firstly, the original positioning error in the navigation data was calculated using the method of this paper. Then we set the constant positioning error and the orientation error in the period when the measuring vehicle passes through the area where the control plane is laid, and the positioning error was calculated again using the above method. The positioning error at this time include the original positioning error and the set constant value positioning error. Finally, after removing the positioning error, compare it with the set constant error.

Experimental statistics are shown in Table 3. Setting different values and different positive and negative positioning errors means that the positioning error obtained by our method is different from the simulated constant positioning error in the millimeter range. It can be seen from Equation (3) that due to the influence of the attitude error, the position corresponding to each point changes when the coordinate system is rotated, so the positioning error obtained in this paper is different from the simulation constant positioning error. With the increase in attitude error, the difference between the positioning error obtained by the method and the simulated constant positioning error is gradually increasing. However, when the attitude error increases to 0.010 degrees, the difference is still in the millimeter range, which has little effect on the final solution.

### 4.3. Positioning Error Compensation Experiment

**Figure 9:**
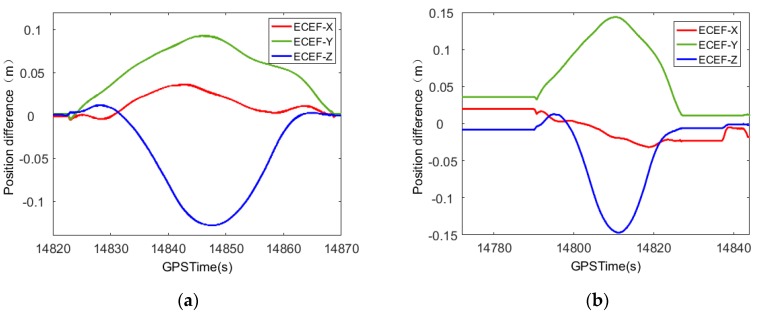
Position error curve of satellite loss of lock in the experimental area of J1 teaching building. (**a**) loss of lock for 90 s; (**b**) satellite loss of lock for 180 s.

First, the original data of GNSS was processed. The GNSS information for the experimental area of the J1 teaching building was deleted for 90 s and 180 s, thus causing an artificial loss-of-lock. The difference between the smoothed loss-of-lock data and the normalized unlocked data is shown in Figure 9. When the satellite signal experienced loss-of-lock for 90 s, the positioning error reached 0.13 m in the ECEF-Z direction and 0.09 m in the ECEF-Y direction. When the satellite loss-of-lock time increases to 180 s, the maximum positioning error in the ECEF-Z direction was 0.15 m and in the ECEF-Y direction it was 0.15 m. Since these data come from the calibration data of mobile measurement, the measuring car needs to be stationary for a period of time before and after passing through the control plane area, thus the inertial navigation error accumulates slowly. Therefore, the positioning error is still on the decimeter level when the satellite experiences loss-of-lock for 180 s.

**Figure 10:**
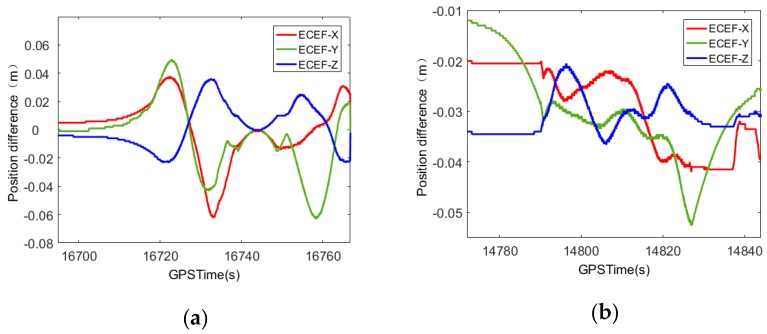
Compensated position error curve in the experimental area of J1 teaching building. (**a**) Satellite loss of lock for 90 s; (**b**) satellite loss of lock for 180 s.

The positioning error obtained after compensation is shown in Figure 10. When the satellite experiences loss of lock for 90 s, the compensated positioning error ECEF-X direction is reduced from 0.03 m to 0.01 m, and in the ECEF-Y direction it is reduced from 0.09 m to 0.04 m. The ECEF-Z direction is reduced from 0.13 m to 0.02 m. When the satellite experiences loss of lock for 180 s, the compensated positioning error ECEF-X direction is reduced from 0.03 m to 0.02 m and in the ECEF-Y direction it is reduced from 0.15 m to 0.02 m, while the ECEF-Z direction is reduced from 0.15 m to 0.01 m. It can be concluded that with the increase in the time of satellite loss of lock, the compensation effect of this method becomes more obvious.

**Figure 11:**
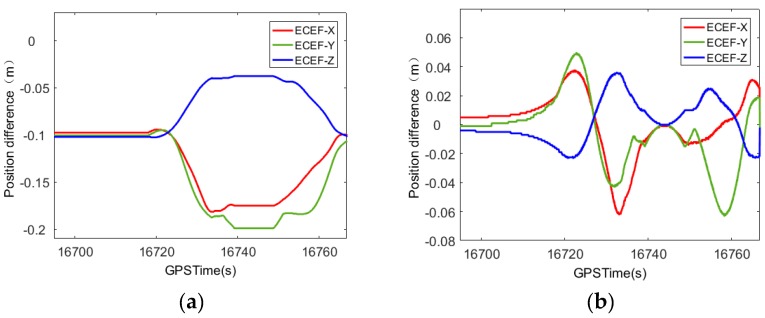
The satellite loss of lock 180 s positioning error compensation before and after the graph in the experimental area of restaurant. (**a**) Satellite loss of lock for 180 s; (**b**) satellite loss of lock 180 s compensation.

In addition, in order to verify the reliability of the method, the 180 s GNSS observation information of the restaurant experiment area was deleted, thus causing an artificial loss-of-lock. The difference between the data positioning error before and after the GNSS loss-of-lock compensation and the normal unlocked data is shown in Figure 11.

The compensated positioning error in the ECEF-X direction is reduced from 0.15 m to 0.02 m, and in the ECEF-Y direction it is reduced from 0.21 m to 0.03 m. The ECEF-Z direction is reduced from 0.13 m to 0.01 m. It can be seen that the method has good repeatability and can provide useful reference for practical engineering applications.

In order to visually show the compensation effect of laser point cloud positioning accuracy. The navigation data before and after the compensation are merged with the point cloud data measured by the laser scanner, and the coordinates of the laser foot point in the ECEF coordinate system are obtained. Then the value is plugged into the corresponding equation of the control plane to calculate the residual of each laser foot point, as follows:

(9) $$\omega_{i}=\left(ax_{i}+by_{i}+zc_{i}\right)-d,$$

The residual means square error statistics of residues before and after compensation in each plane are given in Table 4 and Table 5. The residual means square error of residues of seven planes are shown in Table 6 and Table 7. It is easy to see that the accuracy of the laser point cloud after compensation has been significantly improved.

**Figure 12:**
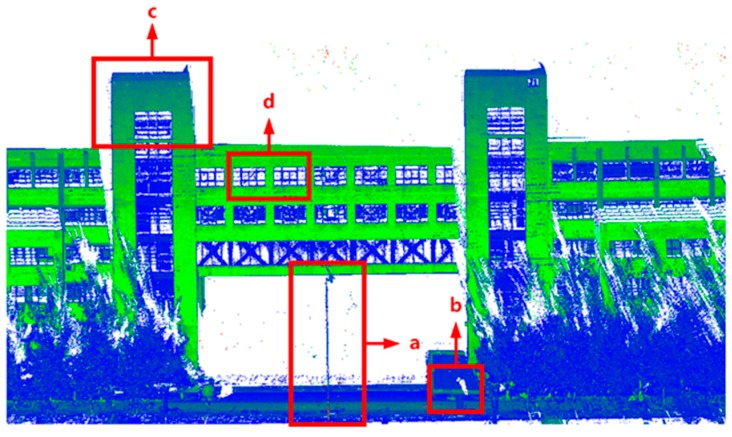
3D point cloud image of the experimental area.

**Figure 13:**
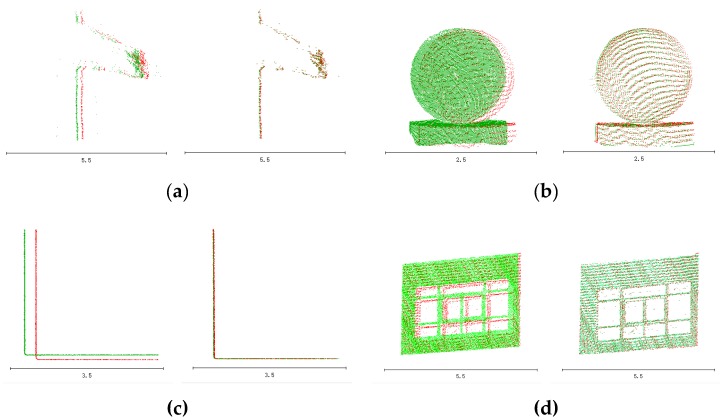
(**a**) The same proportion of the national flag point cloud; (**b**) the same proportion of the stone ball top view point cloud; (**c**) the same proportion of the JIL and SZSF plane point cloud top view point cloud; (**d**) the same proportion of the window point cloud.

Figure 12 is a three-dimensional point cloud image of the experimental area. a, b, c, and d marked in this figure corresponds to those in Figure 13.

Figure 13 shows some details of the three-dimensional point cloud map of the experimental area. Among them, the red point cloud indicates the laser point cloud when GNSS is not experiencing loss-of-lock. The green laser point cloud on the left indicates the laser point cloud before compensation, and the green laser point cloud on the right indicates the compensated laser point cloud. The bottom line in the figure is the size of the scale, which is used to show that the scale of the laser point cloud before and after compensation is the same. From the above experiment, it can be seen that the point cloud before compensation does not coincide with the point cloud calculated normally, and the point cloud compensated for by the method in this paper has an extremely obvious effect of overlapping with the point cloud calculated normally.

It can be seen from the conclusion of the whole experiment that the positioning error compensation method of the mobile measurement system, based on the plane control can effectively reduce the positioning error of the integrated navigation after the loss-of-lock. In addition, the accuracy of the compensated laser point cloud has been significantly improved.

## 5. Conclusions

In this paper, by analyzing the POS error characteristics of satellites in the unlocking state of GNSS, a method of positioning error compensation for mobile measurement systems based on plane control is proposed. The method can calculate the positioning error at an intermediate point and compensate for the entire GNSS loss-of-lock period. This not only overcomes the problem of feature point extraction and precision guarantee, but also improves the work efficiency, at the same time obtaining accurate track information. In our study, not only by comparing the accuracy of the moving measurement system before and after compensation, but also by comparing the positioning error before and after compensation and the accuracy of the mobile measurement system, we prove that this method is a reliable and effective.

In the process of solving the positioning error, when all the point cloud data are used for solving, the solving speed is slow due to the data volume being so large. In this study, the positioning error obtained may not be the optimal solution because only partial points are used to solve the solution. So, it is necessary to further study how to make full use of data to improve the accuracy of the solution. The idea behind this method can also be extended to other features. In future work, we will study the positioning error compensation of mobile measurement systems based on wider features, such as surface features suitable for tunnels.

## Figures and Tables

**Table 1:** Position and attitude error data after GNSS/INS smoothing solution.

Time	ECEF-X (m)	ECEF-Y (m)	ECEF-Z (m)	Roll (deg)	Pitch (deg)	Yaw (deg)
1-min	0.158	0.059	0.033	0.0045	0.0016	0.0011
3-min	0.400	1.005	0.210	0.0042	0.0062	0.0065

**Table 2:** Technical parameters.

Sensor	Technical Parameters	
Laser scanner (Z+F PROFILER 9012)	Laser class	1
	Ambiguity distance (m)	≤0.001
	Angular resolution (deg)	0.0088°
POS(SPAN-LCI)	Position Accuracy/Vertical Position Accuracy (m)	0.01/0.015
	Pitch/Roll Angle Accuracy (deg)	0.005
	Heading Angle Accuracy (deg)	0.008

**Table 3:** Simulation experiment results.

Simulation Constant Error						This Paper Obtained the Positioning Error		
ECEF-X (m)	ECEF-Y (m)	ECEF-Z (m)	Roll (deg)	Pitch (deg)	Yaw (deg)	ECEF-X (m)	ECEF-Y (m)	ECEF-Z (m)
0.05	0.05	0.05	0.005	0.005	0.005	0.0492	0.0488	0.0498
0.10	0.10	0.10	0.005	0.005	0.005	0.0992	0.0988	0.0998
0.15	0.15	0.15	0.005	0.005	0.005	0.1492	0.1488	0.1498
0.05	0.05	0.05	0.008	0.008	0.008	0.0487	0.0481	0.0504
0.05	0.05	0.05	0.010	0.010	0.010	0.0484	0.0476	0.0495
0.05	0.05	0.05	−0.005	−0.005	−0.005	0.0508	0.0512	0.0502
−0.05	−0.05	−0.05	−0.005	−0.005	−0.005	−0.0492	−0.0488	−0.0498

**Table 4:** Compensation for satellite loss of lock of 90 s.

Plane	Before Compensation (m)	After Compensation (m)
EJ1E	0.083	0.010
J1D	0.030	0.009
J1DN	0.026	0.009
J1L	0.123	0.010
J1M	0.078	0.008
J1R	0.044	0.016
SZSF	0.098	0.006

**Table 5:** Compensation for satellite loss of lock of 180 s.

Plane	Before Compensation (m)	After Compensation (m)
EJ1E	0.092	0.004
J1D	0.074	0.015
J1DN	0.069	0.015
J1L	0.182	0.005
J1M	0.106	0.005
J1R	0.062	0.014
SZSF	0.095	0.014

**Table 6:** Compensation for satellite loss of lock of 90 s.

Check the Number of Faces	Before Compensation (m)	After Compensation (m)
7	0.073	0.011

**Table 7:** Compensation for satellite loss of lock of 180 s.

Check the Number of Faces	Before Compensation (m)	After Compensation (m)
7	0.110	0.011

## References

[1] Wan R., Huang Y., Xie R., Ma P. (2019). Combined Lane Mapping Using a Mobile Mapping System. Remote Sens..

[2] Cui T., Ji S., Shan J., Gong J., Liu K. (2017). Line-Based Registration of Panoramic Images and LiDAR Point Clouds for Mobile Mapping. Sensors.

[3] Nivedita S., Sudhagar N., Scott O. (2016). Development of mobile mapping system for 3D road asset inventory. Sensors.

[4] Borja R.C., Silverio G.C., Celestino O., Maria C.A. (2015). An approach to detect and delineate street curbs from MLS 3D point cloud data. Autom. Constr..

[5] Fryskowska A., Wroblewski P. (2018). Mobile laser scanning accuracy assessment for the purpose of base-map updating. Geod. Cartogr..

[6] Handel P., Ohlsson J., Ohlsson M. (2014). Smartphone-Based Measurement Systems for Road Vehicle Traffic Monitoring and Usage-Based Insurance. IEEE Syst. J..

[7] Yu M.J. (2012). INS/GPS integration system using adaptive filter for estimating measurement noise variance. IEEE Trans. Aerosp. Electron. Syst..

[8] Puente I., González-Jorge H., Martínez-Sánchez J., Arias P. (2013). Review of mobile mapping and surveying technologies. Measurement.

[9] Barber D., Mills J., Smith-Voysey S. (2008). Geometric validation of a ground-based mobile laser scanning system. ISPRS J. Photogramm. Remote Sens..

[10] Peng K.Y., Lin C.A., Chiang K.W. (2012). The performance analysis of an AKF based tightly-coupled INS/GPS integrated positioning and orientation scheme with odometer and non-holonomic constraints. Int. Arch. Photogramm. Remote Sens. Spat. Inf. Sci..

[11] Angrisano A., Gaglione S., Gioia C. (2013). Performance assessment of GPS/GLONASS single point positioning in an urban environment. Acta Geod. Geophys. Hung..

[12] Klein I., Filin S., Toledo T. (2011). Vehicle constraints enhancement for supporting INS navigation in urban environments. Navigation.

[13] Vagle N., Broumandan A., Lachapelle G. (2018). Multi-Antenna GNSS and Inertial Sensors/Odometer Coupling for Robust Vehicular Navigation. IEEE Internet Things J..

[14] Broumandan A., Lachapelle G. (2018). Spoofing detection using GNSS/INS/Odometer coupling for vehicular navigation. Sensors.

[15] Gong X., Zhang R., Fang J. (2013). Application of unscented R–T–S smoothing on INS/GPS integration system post processing for airborne earth observation. Measurement.

[16] Shi B., Lu X., Chen Y.F. (2012). Improving position and attitude precision of GPS/INS by applying EKF smoothing algorithm. J. Geomat. Sci. Technol..

[17] Qiu R., Cheng Y. (2015). The interpolation application of interval quartering algorithm of singular spectrum analysis iterative in GPS coordinate time series. J. Geod. Geodyn..

[18] Liu S., Sun F., Li H. (2015). Forward-backward-smoothing algorithm with application to tightly coupled PPP/INS data post-processing. J. Chin. Inert. Technol..

[19] Liu H., Nassar S., El-Sheimy N. (2010). I Two-Filter Smoothing for Accurate INS/GPS Land-Vehicle Navigation in Urban Centers. IEEE Trans. Veh. Technol..

[20] Nassar S., Niu X., El-Sheimy N. (2007). Land-Vehicle INS/GPS accurate positioning during GPS signal blockage periods. J. Surv. Eng..

[21] Takai S., Date H., Kanai S., Niina Y., Oda K., Ikeda T. (2013). Accurate registration of MMS point clouds of urban areas using trajectory. ISPRS Ann. Photogramm. Remote Sens. Spat. Inf. Sci..

[22] Schaer P., Vallet J. (2016). Trajectory adjustment of mobile laser scan data in GPS denied environments. ISPRS Ann. Photogramm. Remote Sens. Spat. Inf. Sci..

[23] Han J.Y., Chen C.S., Lo C.T. (2013). Time-Variant registration of point clouds acquired by a mobile mapping system. IEEE Geosci. Remote Sens. Lett..

[24] Kai-Wei C., Trung D.T., Liao J.K. (2012). On-Line smoothing for an integrated navigation system with Low-Cost MEMS inertial sensors. Sensors.

[25] Sharkarji C.M. (1998). Least-Squares fitting algorithms of the NIST algorithm testing system. J. Res. Natl. Inst. Stand. Technol..

